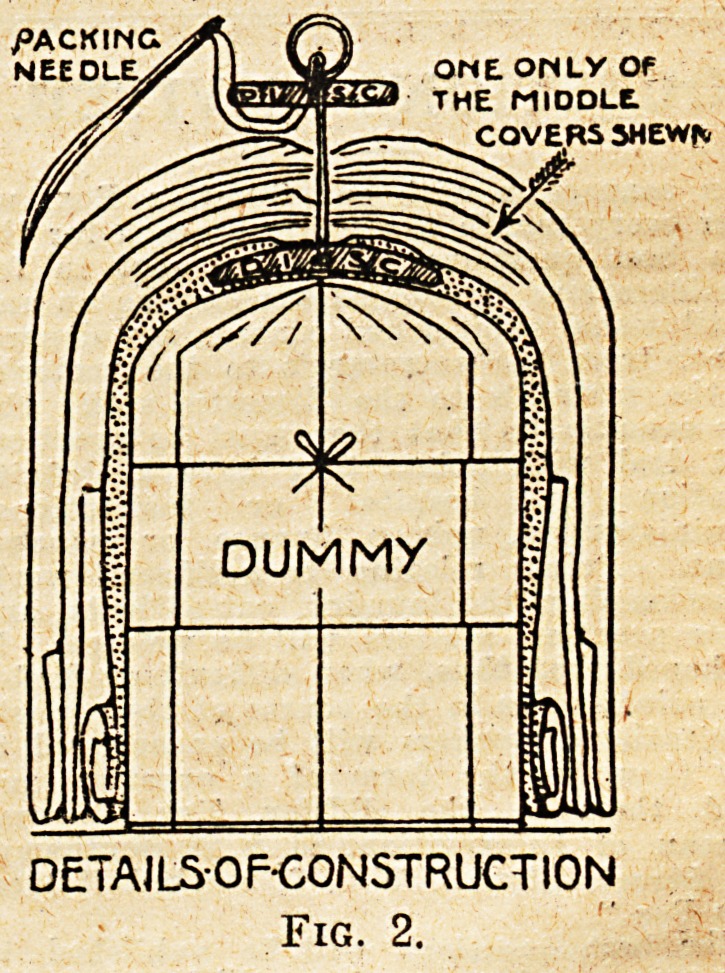# The Institutional Worker

**Published:** 1918-05-18

**Authors:** 


### THE INSTITUTIONAL WORKER

**Being a Special Supplement to "The Hospital"**

### OUR BUREAU OF INFORMATION.

**Rules for Correspondents.**

1. Every letter must be accompanied by the coupon to be cut from the back cover (inside page) of The Hospital, current issue, and must contain the name and address of the correspondent with pseudonym for publication if desired. Replies by post cannot be given save under exceptional circumstances at the Editor's discretion.

2. Letters from Approved Homes in reply to special needs published in the Bureau should state terms and full particulars, and be sent prepaid under cover to the Editor of the Bureau with name written across coupon for identification.

3. Proprietors of Homes which have not yet been entered on the List of Approved Homes, but have spare accommodation likely to suit special needs, are invited to write for an application form for registration. The fee for registration, which includes two announcements of the Home in the Bureau and other privileges, is 10s.

4. All communications to be addressed to the Editor of The Hospital, 28 Southampton Street, Strand, London, W.C. 2, and marked "Bureau of Information."

### INSTITUTION FACTS AND FIGURES.

### FIRELESS COOKERY.

### NESTED COVERS: THE HAY-BOX SUBSTITUTE.

A great deal has been written in these columns on the subject of fireless cookery. Mr. Cornwell Round, M.R.C.S., has recently applied for a patent for a very simple device to take the place of the hay-box, for, as he states, it is not easy in these days to get either a box or the requisite hay for the purpose. Pending the time when this device is placed on the market, Mr. Round suggests that, in the interests of national economy,

Fig. 1.

householders and institutional housekeepers should make for themselves the simple contrivance, which he terms a "nested cover." It can be constructed entirely from newspaper. In form it has the appearance of the straw beehive. It should be large enough to slip over the cooking utensil, which, he suggests, should take the form of a three-quart drop-handle camp saucepan.

The method of making a nested cover is as follows: Cut out a number of discs from pieces of newspaper (a tea-plate or saucer will serve as a pattern). Take a carpet needle, and through the centre of about a dozen of the discs run a string two feet long, the two ends of which are knotted together, drawing the discs down on to the knot; paste a few discs on to the knot so as to hide it. Then around a dummy a little larger than the cooking vessel wrap a piece of newspaper folded lengthwise, and paste the side edges together so as to form a cylinder, taking care not to paste on to the dummy. Place the discs already threaded on the top of the dummy, keeping the string and needle uppermost, and crumple over them the ends of the folded newspaper, which should be brought close together. Thread several more discs on the string, put some paste on them and on the string, and pass them down on top of the crumpled paper so as to form a complete comparatively air-tight cover. Repeat the process of wrapping with newspaper, pasting the edges, crumpling the ends, and placing pasted discs until there are thirty or more similar discs or covers superimposed on one another. Throughout the process it should be remembered to paste lightly only. The object is not to make pasteboard, but to form air-spaces between each layer of paper, so as to prevent heat radiation.

To finish the cover thread some discs on the needle, pass it through an ordinary curtain-ring (see fig. 2), bring it back through the same hole in the discs, draw the string tightly so that the ring rests firmly on the discs, cut off the needle, pass the ends of string several times round the double string underneath the discs, and tie in a strong knot. Paste down the disc.

If no shape or dummy is available of the size required, the saucepan can be used, tying some paper round it to increase the diameter so that the nested cover, when complete, can be slipped easily over the saucepan.

A pad of paper upon which to rest the saucepan before placing the nested cover over it is desirable. This can be made by sewing together a number of discs of paper the same size as the bottom of the saucepan. Sew four buttons, rolls of paper, or other non-heat-conducting objects near the circumferences at equal distances on both sides of this paper pad, upon which the saucepan can rest. By using

Fig. 2.

these buttons only a small surface will be exposed to direct conduction of heat. The nested cover can be used in the same way as the hay-box.

### MAY QUESTIONS.

### (1) The Organisation of a Creche in a Munition Factory.

**A large firm employing about 2,000 women workers, many of whom are married, are anxious to start a creche or day-nursery, for the benefit of the married women, who will be enabled to leave their babies there during working hours. Describe fully the equipment and accommodation required for about fifty babies, and the estimated cost for one year's running. There is already a good welfare department—with two welfare supervisors and two nurses—and it is estimated that the latter could help for a short time each day, and the supervision of the creche would come directly from the head welfare worker.**

### (2) The Protection of Linen in Bedridden Cases.

**In certain Poor-Law infirmaries ointment and oil mixtures are frequently in use for the treatment of backs and pressure point in bedridden cases, especially when wasting diseases are evidenced. What can be recommended as an economical procedure in the matter of personal and bed linen, bearing in mind the effect of grease and oil on linen?**

#### RULES.

**The following rules must be observed:—**

**1. Contributions must be written on one side of the paper. Brevity and terseness are desirable features. The MSS. must bear the name and address of the sender and be accompanied by coupon to be cut from the back cover (inside page) of the current issue of THE HOSPITAL. A pseudonym must be chosen if the name is not to be published.**

**2. Contributions must be addressed to the Editor of THE HOSPITAL, Institutional Worker Supplement, 28 & 29 Southampton Street, Strand, London, W.C. 2, should reach him before the end of the current month, and be marked in the left-hand corner "Facts and Figures."**

**A minimum payment of Five Shillings will be made for each published answer.**

### QUESTIONS INVITED.

**In connection with our Question Box, we offer every month five shillings each for the two best questions which are sent in for consideration. The questions may be on any institutional subject and concern any department, from the secretary's to the porter's, or the matron's to the domestic staff; the one essential is that they must be practical and deal with points that have a definite relation to hospital or institutional administration, upkeep, finance, or management. Points relating to artificers' work, housekeeping, the laundry, out-patients, and other practical matters will be welcome. It is hoped that thus, when difficult or doubtful points arise in the course of their work, institutional workers will be encouraged to put them in the form of a question and send them to the Editor, THE HOSPITAL Bureau of Information, 28 & 29 Southampton Street, Strand, W.C. 2, marked "Question Box."**

**The best questions will be published in due course and our readers will be given the opportunity of answering them. Thus all institutional workers may be able to cooperate with the view of helping the work and smoothing the difficulties of each other. In short, we want the Question Box of the *Institutional Worker* to be freely used by every worker habitually.**

### ENQUIRIES AND ANSWERS.

### *THE SICK AND IN NEED.*

#### Orthopædic Hospital.

OUT-PATIENTS are seen at the Royal National Orthopædic Hospital, 234 Great Portland Street, W., at 1.30 P.M. every day except Saturday and Sunday. Full inquiry will be made as to the means of the patient and the suitability of the case for hospital treatment.—VIC.

### *EMPLOYMENT AND TRAINING.*

#### Training Dispensers in Hospitals.

YES, it would be possible to train dispensing pupils in an average-sized hospital provided that the accommodation of the dispensary were suitable and the needful appliances were available. We would refer you to an interesting article on this subject which appeared in *The Institutional Worker* published on November 27, 1915.—F. W. H.

#### Irish Poor-Law Charge Nurse.

YOUR training at the hospital you mention, together with your midwifery training, should qualify you to obtain a post as charge nurse in a Poor-Law infirmary.—IRISH MOLLY.

### *WAR MATTERS.*

#### Disused V.A.D. Uniforms.

WITH a view to prevent waste and to enable some of the new members to join on easier terms, the Committee of the British Red Cross Society have arranged, with the approval of Devonshire House, to allow retiring members of the V.A.D. Section to advertise their discarded uniforms in the columns of their official journal, the *Red Cross.* The following rules must be observed:—(1) Advertisements will only be received through a commandant, whose address and number of detachment will be inserted in the advertisement. (2) All correspondence as to sales and purchases must pass between the commandants and intending purchasers direct, and not through the office of the *Red Cross*, which accepts no responsibility to either party in respect of the transactions. (3) Only official uniforms will be the subject of advertisement. Should the demand warrant it, this arrangement will be made permanent and a small charge fixed.

#### The Scarlet Efficiency Stripe and War Service Bar.

A SCARLET "Efficiency Stripe" is given to nursing V.A.D. members and special service probationers in military hospitals after thirteen months' consecutive service, if they are specially recommended for this distinction by the matron The stripe is worn on the right arm above the elbow, and on the indoor uniform only. This stripe is not issued from Devonshire House. It is a military award, and can only be obtained from the matrons of the military hospitals. A white "War Service Bar" is issued by the Joint Committee of the British Red Cross Society and the Order of St. John of Jerusalem for length of service.—TERRY.

### *THE HOUSEKEEPERS' DEPARTMENT.*

#### An Economical Syrup Sauce.

INSTITUTION housekeepers may welcome the following recipe, which will enable them to make 1 lb. of treacle or syrup go as far as 2 lb. in these days of scarcity. The ingredients used are: 1 tablespoonful cornflour, 1 teaspoonful ground ginger, 1 pint cold water, 1 pint syrup or treacle, a few drops lemon juice or essence of lemon.

*Method.*—Out of the pint of water take sufficient to mix the cornflour and ginger; boil the remainder and add to the cornflour; pour back into the saucepan, boil for a few minutes, add the syrup or treacle and bring to boiling-point; then add the flavouring.

The result is a delicious sauce which will make the institution suet-pudding a popular and appetising sweet.

#### Recipe for Potato Bread.

THE recipe you ask for, we believe, is the one which has been circulated by Mr. Henry Gandy, County Director of Cumberland West, amongst the Cumberland V.A.D. hospitals. It is: 9 lb. flour, 3 lb. boiled and mashed potatoes, $1\frac{1}{2}$ oz. balm (yeast), 5 pints water, salt to taste. Mix the potatoes while still warm in a basin with the yeast, two or three handfuls of flour, and sufficient water to form a sponge. Leave this sponge to rise for fifteen minutes. Then knead it into the remainder of the flour, adding the rest of the water and a little salt. Allow this to rise for one or two hours, and then bake.—W. D. C.

#### Stained Table-Linen.

THE simplest method of removing tea and coffee stains from linen is to place the stained part in cold water at once, afterwards pouring boiling water over it. Fruit and wine stains can be removed by rubbing the linen well with common salt and pouring boiling water over it. The rubbing should be continued until the stain has disappeared. This can best be done by stretching the linen over a basin. Soap should not be applied to a stain, for it tends to fix it.—J. W.

### APPROVED HOMES.

WE have pleasure in adding the following to our List of Approved Homes:—WHITCHURCH, SALOP, Miss E. Acland-Nurse, 3 Edgeley Road. Home for permanent invalid, medical, surgical, maternity, massage, nerve, convalescing. Two patients only taken. Terms from three guineas a week for ordinary cases, four guineas for maternity and nerve.

## Figures and Tables

**Fig. 1. f1:**
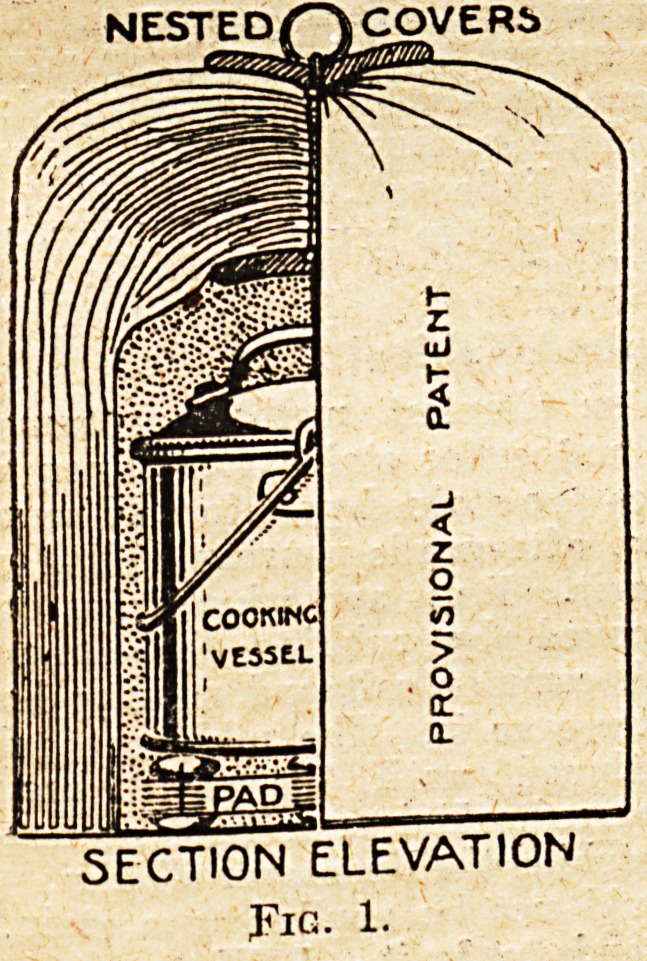


**Fig. 2. f2:**